# Reactive Nanoparticles Derived from Polysaccharide Phenyl Carbonates

**DOI:** 10.3390/molecules26134026

**Published:** 2021-07-01

**Authors:** Martin Gericke, Katja Geitel, Cornelia Jörke, Joachim H. Clement, Thomas Heinze

**Affiliations:** 1 Institute of Organic Chemistry and Macromolecular Chemistry, Friedrich-Schiller-University of Jena, Humboldtstr 10, 07743 Jena, Germany; 2 Department of Hematology and Medical Oncology, Jena University Hospital, Am Klinikum 1, 07747 Jena, Germany; katja.geitel@uni-jena.de (K.G.); cornelia.joerke@med.uni-jena.de (C.J.); joachim.clement@med.uni-jena.de (J.H.C.)

**Keywords:** polysaccharide derivatives, homogeneous synthesis, nanoparticles, xylan derivatives, biocompatibility, dye functionalization

## Abstract

Polysaccharide (PS) based nanoparticles (NP) are of great interest for biomedical applications. A key challenge in this regard is the functionalization of these nanomaterials. The aim of the present work was the development of reactive PS-NP that can be coupled with an amino group containing compounds under mild aqueous conditions. A series of cellulose phenyl carbonates (CPC) and xylan phenyl carbonates (XPC) with variable degrees of substitution (DS) was obtained by homogeneous synthesis. The preparation of PS-NP by self-assembling of these hydrophobic derivatives was studied comprehensively. While CPC mostly formed macroscopic aggregates, XPC formed well-defined spherical NP with diameters around 100 to 200 nm that showed a pronounced long-term stability in water against both particle aggregation as well as cleavage of phenyl carbonate moieties. Using an amino group functionalized dye it was demonstrated that the novel XPC-NP are reactive towards amines. A simple coupling procedure was established that enables direct functionalization of the reactive NP in an aqueous dispersion. Finally, it was demonstrated that dye functionalized XPC-NP are non-cytotoxic and can be employed in advanced biomedical applications.

## 1. Introduction

Nanomaterials possess unique properties that are different compared to the individual molecule or the bulk compound due to their nm-scaled size [1,2]. They can be manufactured from various inorganic, organic, and hybrid compounds by using different top-down and bottom-up methods [3,4]. Nanoparticles (NP) derived from organic polymers are highly versatile because their surface chemistry as well as their overall physical and biological properties can be tuned to a vast degree by taking advantage of the possibilities provided by modern organic chemistry. They are studied intensively for therapeutic drug delivery purposes [5,6]. If modified with dyes (fluorescence, UV/Vis, NIR) and/or specific bio-affinity ligands (e.g., antibodies, receptors), polymer-based NP can also be employed for in vitro/in vivo imaging and sensing [7,8]. In the context of these biomedical applications, nanomaterials derived from biopolymers such as polysaccharides (PS) possess inherent advantages because of their native biocompatibility and biodegradability [9,10]. A convenient and versatile approach for preparation of PS-NP is the self-assembling of hydrophobically modified PS derivatives [11]. However, a key challenge for these materials is the introduction of functionalities (e.g., dyes, drugs, stimuli responsive groups, (bio-) affinity ligands) that are required for a specific application but not present in the original PS backbone.

A viable approach for obtaining functional PS-NP is the chemical modification of the PS backbone with the respective functionalities prior to the self-assembling step. It is particularly suitable if the substituents (e.g., drug molecules) are hydrophobic themselves and can thus initiate the self-assembling procedure without the need for additional hydrophobic groups [12,13]. This “bulk modification” approach can yield high loading efficiencies. However, it is less suitable for functionalities that are only active at the NP surface, such as receptors, bio-affinity ligands, and fluorescent probes. Surface modification of NP requires highly efficient coupling reactions that work under aqueous conditions because the substrates are often rather expensive and sensitive, especially in the case of biomolecules. In this context, PS-NP with activated ester groups have been developed recently that showed high coupling efficiencies for functional molecules with amino groups [14]. Nevertheless, new approaches towards reactive PS-NP are in constant demand. In particular, the number of reaction steps for the synthesis of the particle forming PS derivatives should be kept to a minimum.

PS derivatives with phenyl carbonate substituents derived from cellulose and xylan were found to be very versatile intermediates that can react with a broad variety of molecules that carry nucleophilic amino groups [15,16]. At the same time, nano self-assembling of some PS derivatives with carbonate substituents (aryl and alkyl) has been reported [17,18]. It is reasonable to assume that carbonate substituents can fulfill a dual purpose to provide an easy access to reactive PS-NP. The goal of this work was to comprehensively study the nano self-assembling of hydrophobic PS phenyl carbonate derivatives and to verify or disprove three hypothesis: (i) cellulose phenyl carbonates (CPC) and xylan phenyl carbonates (XPC) are suitable for the preparation of PS-NP, (ii) the PS-NP obtained possess reactive phenyl carbonate groups on the surface that can be converted with amines under aqueous conditions, and (iii) the reactive PS-NP are suitable for biomedical applications.

## 2. Results and Discussion

### 2.1. Evaluation of the Self-Assembling Behaviour

**Figure 1:**
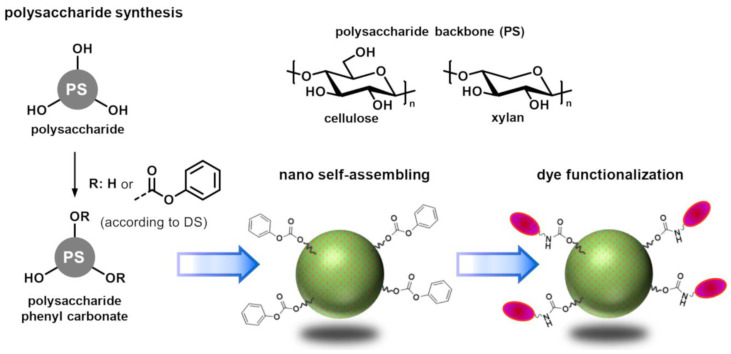
Scheme for the synthesis of polysaccharide phenyl carbonates as well as for the subsequent formation and dye functionalization of reactive nanoparticles.

A series of polysaccharide (PS) phenyl carbonates was synthesized to evaluate the nanoparticle (NP) formation comprehensively (Figure 1). Homogeneous synthesis procedures were employed in order to tune the degrees of substitution (DS) over a broad range [18,19,20]. Cellulose and xylan were chosen as starting materials because of their different molecular structures. Hydrophobic cellulose- and xylan derivatives (mostly esters) have been proven previously to form PS-NP with high application potential. However, they were not yet compared within the frame of one comprehensive study in order to evaluate the effect of the PS backbone on the self-assembling behavior [11]. Both PS feature two secondary hydroxyl groups in positions 2 and 3 but cellulose features an additional -CH_2_-OH moiety in position 6. It was of great interest to evaluate the potential influence on the nano self-assembling process. The PS-NP in this study were prepared by a dialysis approach [21]. Cellulose phenyl carbonates (CPC) and xylan phenyl carbonates (XPC) were dissolved in *N*,*N*-dimethylacetamide (DMA) with different mass concentrations and the organic solutions were dialyzed against an excess of water. The aqueous particle dispersions obtained were characterized by dynamic light scattering (DLS) to determine the particle size and its distribution (Table 1).

Dialysis of CPC solutions resulted in rapid transition from optical clear solutions to turbid aqueous dispersions. Similar behavior has been reported previously for the nanoprecipitation of a variety of hydrophobic cellulose derivatives (in particular esters) [22,23,24]. Thus, it can be concluded that self-assembling of CPC occurred. However, the dispersions obtained from these particular cellulose derivatives were not stable and sedimented over the course of several days (see Supplementary Materials, Figure S1). The particle size distribution was determined directly after finishing the particle formation procedure, i.e., 24 h after starting the initial dialysis, by dynamic light scattering (DLS; Table 1). Nano self-assembling of cellulose derivatives is promoted by hydrophobic interaction of the substituents, which results in the formation of PS-NP with particles sizes roughly in the range of 50–500 nm [11]. For CPC with a DS of 3.0, i.e., complete conversion of all cellulosic hydroxyl groups, formation of aggregates was observed with sizes above 1 µm rather than NP (**CPC-NP 1**–**5**). The polydispersity indices (PDI) determined for these materials were comparably high, which is an indication for a broad distribution of particle sizes, i.e., an uncontrolled aggregation process. A similar behavior has been described for fully acetylated cellulose [22]. It has been proposed for nanoprecipitation of polymers in general that a certain balance between hydrophobic and hydrophilic moieties is favorable [25].

**Figure 2:**
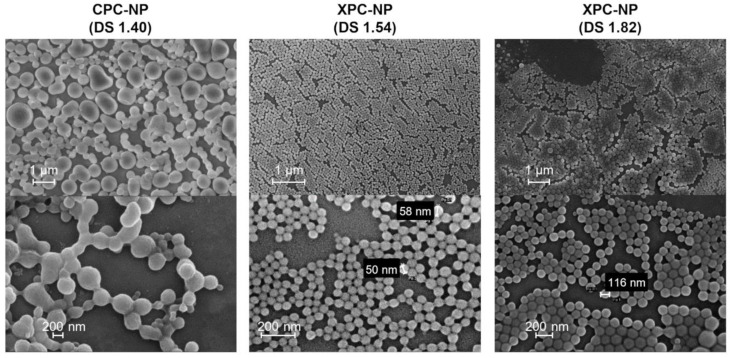
Scanning electron microscopy images of nanoparticles (NP) obtained by self-assembling of cellulose phenyl carbonates (CPC) and xylan phenyl carbonates (XPC) with different degrees of substitution (DS).

Decreasing the DS value to 1.90 (**CPC-NP 6**–**10**) and 1.40 (**CPC-NP 11**–**15**) yielded smaller particles with sizes in the range of about 400 to 1800 nm, which is still rather high in comparison to actual nm-sized PS-NP obtained from other hydrophobic PS derivatives. The particles had irregular shapes and a broad distribution in size (Figure 2). These findings are contrary to previous studies that showed very good NP formation properties for many hydrophobic cellulose ester and carbamates with high to intermediate DS values [11]. However, CPC with a comparably low DS of 1.08 formed PS-NP in the expected range of about 200 to 500 nm (**CPC-NP 18**–**20**), which is similar to previously reported PS-NP obtained by dialysis of cellulose derivatives. Further decreasing the DS of CPC to 0.85 had an adverse effect. The particle sizes increased to above 1 µm for most mass concentrations (**CPC-NP 21**–**25**).

**Figure 3:**
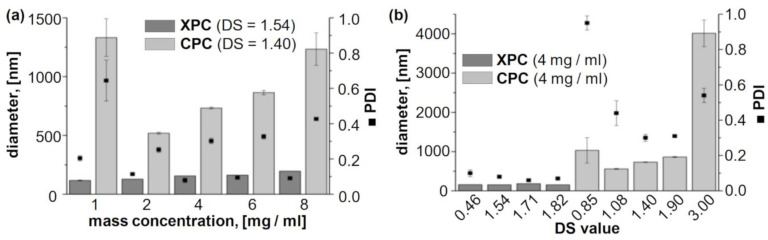
Particle sizes and polydispersity indices (PDI) of nanoparticles obtained by self-assembling of xylan phenyl carbonates (XPC) and cellulose phenyl carbonates (CPC) using a dialysis approach and (**a**) samples with similar degrees of substitution (DS) or (**b**) the same mass concentration for the starting polymer solution.

Self-assembling of XPC by dialysis-induced nanoprecipitation was studied for derivatives with DS values ranging from 0.42 to 1.82 (Table 1). It should be noted in this context, that xylan features only two hydroxyl groups (i.e., DS_max_ = 2.0 for XPC) as opposed to three hydroxyl groups in cellulose (i.e., DS_max_ = 3.0 for CPC). Stable aqueous particle dispersions were obtained for all XPC derivatives used in this study. DLS measurements demonstrated no correlation between particles size and the DS for XPC derivatives (Table 1, Figure 3). However, the mass concentration of XPC in the organic starting solution had a small influence. Independent of the DS value, particles sizes of around 100 to 120 nm were obtained when using 1 mg_XPC_/mL. The particle size consistently increased to values of around 200 to 220 nm when increasing the mass concentration up to 8 mg_XPC_/mL. Thus, it is possible to tune the particle properties of XPC-NP to a certain extent. The PDI was ≤0.2 in almost all cases, which is an indication for a very narrow size distribution and a uniform self-assembling process. Scanning electron microscopy images reveled a regular spherical shape and diameters in the scale of about 50 to 200 nm for XPC-NP (Figure 2). It should be noted here that particles size values of soft polymer NP obtained by DLS (measured on hydrated and potentially swollen NP) and scanning electron microscopy (measured on dried and potentially shrunken NP) are not necessarily identical for the same sample although they are in the same scale range [11]. Furthermore, aqueous XPC-NP dispersions showed no signs of sedimentation even after storage for several months (see Supplementary Materials, Figure S1). Aqueous dispersions of particles derived from CPC derivatives sedimented over the course of several days, probably due to aggregation of smaller NP into larger particles. This self-aggregation could explain the rather high particles sizes observed for the dialysis experiments with CPC derivatives.

To summarize, XPC derivatives showed excellent nano self-assembling over the whole range of DS values tested (0.5 to 1.8) whereas CPC derivatives formed PS-NP only in a small window of DS values (≈1.1). It should be emphasized that this trend holds true only for the particular PS derivatives with aromatic PC substituents used in this study. Other cellulose- and PS derivatives, including ones with aromatic phenyl carbamate, tosylate, benzoate, and phthalate moieties are well suited for the formation of spherical PS-NP [21,26,27,28]. Thus, the present work demonstrates the importance of the molecular structure of PS derivatives on the self-assembling process.

The reason for the unexpected differences of CPC and XPC derivatives regarding their self-assembling behavior is not clear yet. It can be assumed that both types of derivatives initially form nm-scaled particles. CPC-NP further aggregate into larger agglomerates whereas XPC-NP remain stable against sedimentation. This finding cannot be explained by solely considering the amount of hydrophobic groups (DS) or the balance of hydrophobic and hydrophilic groups within the polymer backbone (DS vs. DS_max_). In CPC, the phenyl carbonate moiety is primarily located at the C-6 position due to the higher reactivity of the primary hydroxy group in the homogeneous synthesis [15]. It can be speculated that these phenyl groups are more flexible compared to ones that are attached to secondary positions C-2 and C-3 and that this flexibility enables secondary interaction (e.g., π-π stacking) of the aromatic groups resulting in stronger attraction of the polymer chains and aggregation into larger agglomerates. It might be possible to verify this hypothesis in future studies by using regioselectively modified 2,3-*O*-CPC, which can be synthesized using protecting group strategies [29].

### 2.2. Functionalization of Reactive Nanoparticles

PS-NP have great application potential, e.g., for drug delivery and sensing applications [11]. A key factor in this regard is functionalization of the PS-NP, which can be achieved by three different approaches. Substances such as drugs and dyes can be entrapped physically within the PS-NP matrix by adding these compounds during the nanoprecipitation step [21,30,31]. Another approach is covalent fixation of the desired molecules to the PS backbone [12,13,32]. These substituents could be hydrophobic themselves or might require additional hydrophobic substituents in order to induce nano self-assembling. Both approaches are viable and frequently employed not only for PS-NP but also for many other types of nanomaterials. However, it needs to be considered that incorporating additional substances and/or substituents is likely to affect the self-assembling properties of the hydrophobic PS derivatives. Thus, property tuning can be difficult for these systems. A third possibility is the post-functionalization of PS-NP with the desired components subsequent to the particle formation [14]. The advantage of this approach is that particles can be tailored in advanced and prepared on stock. Moreover, the active groups are only located on the surface, which is preferred for many applications that do not aim for a disintegration of the particles during application. The disadvantage is that the particle formation has to be performed under aqueous conditions, which requires specific coupling techniques to achieve the high conversion efficiencies that are required for expensive drugs, dyes, and biomolecules.

Straightforward nanomaterials should feature reactive groups that are stable under aqueous conditions yet still accessible for highly efficient and selective chemical modification reactions. Thus, high coupling efficiencies can be achieved and the number of process steps (e.g., addition of buffers, centrifugation, redispersing, dialysis) that could result in aggregation of the aqueous PS-NP dispersions is reduced. Some examples for reactive PS-NP have been reported in the literature in which reactive moieties, such as amino-, azido, and activated ester groups, were introduced into different PS in addition to hydrophobic substituents that are required for inducing the self-assembling procedure [14,26,33]. In order to minimize the synthesis efforts, it is desirable to develop systems in which only one substituent fulfills both functions. In this work, it has been demonstrated that phenyl carbonate substituents enable efficient nano self-assembling of xylan-based derivatives. Previously, it has also been reported that XPC derivatives react with a broad variety of amines under homogenous conditions to form functional xylan carbamate derivatives [16]. Thus, it was hypothesized that XPC-NP could be used for a direct coupling of molecules bearing amino groups. The coupling reaction would proceed on the particle surface by conversion of phenyl carbonate groups with amino moieties of primary aliphatic amines and the formation of covalent carbamate linkages (Figure 1). Of particular interest in this regard are biomolecules such as proteins, antibodies, and specific peptide sequences. To verify this hypothesis, conversion of these particles with an amino group containing dye (see Supplementary Materials, Figure S3) was studied.

**Figure 4:**
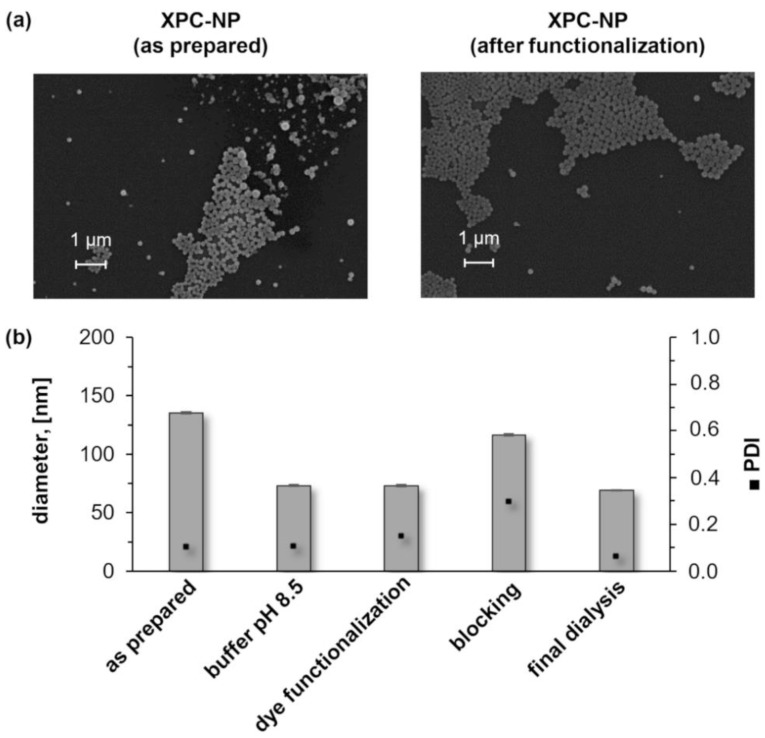
(**a**) Scanning electron microscopy images of nanoparticles (NP) obtained by self-assembling of xylan phenyl carbonate (XPC; degree of substitution of 1.71, initial mass concentration of 4 mg/mL) before and after conversion with amine functionalized dye DY 605 and ethanolamine (blocking reagent). (**b**) Particle sizes and polydispersity indices (PDI) of XPC-NP (degree of substitution of 1.82, initial mass concentration of 4 mg/mL) determined after each individual process step during the functionalization with amine functionalized dye DY 605.

Aqueous XPC-NP dispersions showed a high stability against aggregation and sedimentation. After one month of storage in water a polymer sample was isolated from the particle dispersion by lyophilization. The corresponding FTIR-spectrum showed no significant differences compared to the spectrum of the original XPC derivative (see Supplementary Materials, Figure S2). This is an indication that no hydrolysis of phenyl carbonate groups occurred in water, which is a basic requirement for long-term stability and chemical reactivity. In order to evaluate if XPC-NP are reactive towards amines, aqueous particle dispersion (initial mass concentration of 4 mg/mL) was converted directly with a solution of an amino group functionalized dye (DY 605; see Supplementary Materials, Figure S3). A coupling procedure was devised that was based on the covalent binding of the dye through formation of carbamate linkages and the particle sizes and PDI values were monitored for each individual step (Figure 4). The aqueous XPC-NP dispersion was converted to a buffered system with a pH value of 8.5 to ensure that the amino group of the dye, which was provided as a hydrochloride, was fully deprotonated (i.e., reactive towards the phenyl carbonate groups). No significant changes of the NP were detected. Subsequently, aliquots of aqueous dye solution were added with ratios of 0.2 to 200 nm_dye_ per mg_XPC-NP_. After 24 h of incubation, an excess of ethanolamine (≈1 µL per mg_XPC-NP_) was added as “blocking agent” to ensure that the remaining phenyl carbonate moieties were converted as a precaution for undesired coupling reactions, e.g., with proteins within living organism. Finally, the particle dispersions were dialyzed to remove excess buffer, coupling reagent, and unbound dye. However, it should be noted that even at the highest dye concentration, no apparent discoloration of the deeply colored PS-NP dispersion occurred (see Supplementary Materials, Figure S4) and that the dialysates showed no color or fluorescence either. These findings are clear indications for the covalent immobilization of dye molecules on the surface of XPC-NP. The scanning electron microscopy images and DLS measurements demonstrate that no agglomeration or sedimentation (e.g., induced by covalent cross-linking or by altering the surface charge due to changes of the pH value) occurred during the dye functionalization procedure (Figure 4). The apparent particle size slightly decreased upon addition of the buffer solution, which can be attribute to alteration of the particles solvate layer due to the changes in pH value and ionic strength. Overall, it can be concluded that the hypothesis of this work was verified. NP prepared by self-assembling of XPC derivatives are stable in water yet reactive towards compounds bearing amino groups. Thus, these materials can be employed for a direct coupling, e.g., with dyes, drugs, antibodies, and peptides, for potential biomedical applications.

### 2.3. Bio-Compatibility Studies

**Figure 5:**
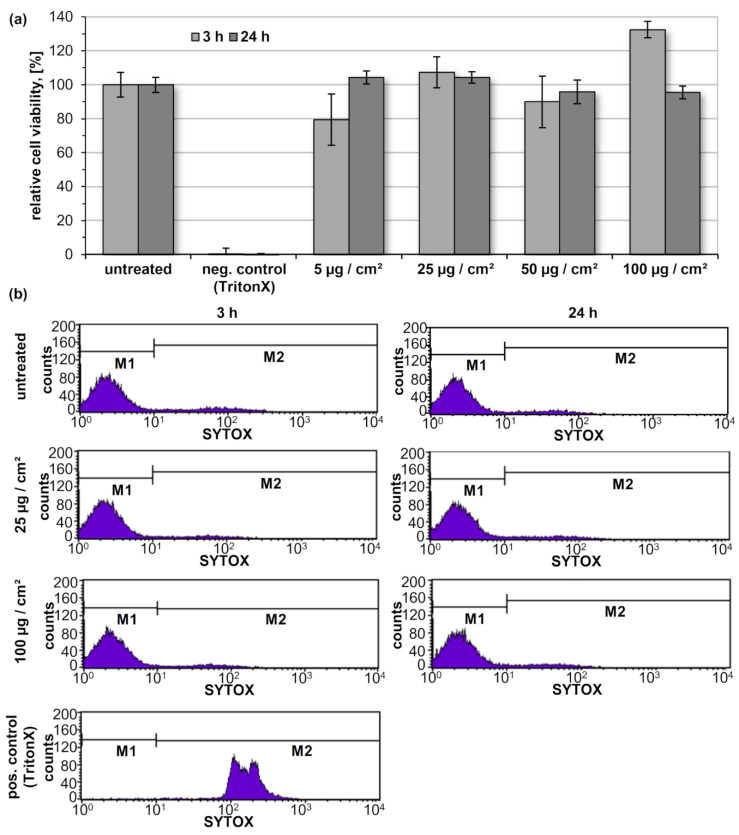
Results of cell viability assay (**a**) and flow cytometry assisted quantification of dead cells (**b**) using the hypopharyngeal carcinoma FaDu cell line after incubation with TritonX-100 (negative control in cell viability assay, positive control in flow cytometry assay) and with different amounts of dye functionalized xylan phenyl carbonate nanoparticles (XPC-NP).

Biocompatibility is a prerequisite for in vitro/in vivo use of PS-NP especially for biomedical applications. Thus, cytotoxicity of XPC-NP was evaluated using aqueous particle dispersions (DS = 1.54; initial mass concentration of 4 mg/mL) after dye functionalization and blocking on the squamous hypopharyngeal carcinoma cell line FaDu. This well-established cell line has been employed before for evaluating the biocompatibility of a broad range of nanomaterials [34]. After an incubation of the surface adherent cells with different amounts of XPC-NP (5 to 100 µg of NP per cm² of cell culture medium) for 3 h and 24 h, no major effect on the viability of FaDu cell cultures was found using the PrestoBlue cytotoxicity assay (Figure 5a). Even in the presence of a high amount of XPC-NP (100 µg/cm²) the viability of the treated cells was close to the untreated control. Thus, the novel PS based NP can be classified as not having cytotoxic potential according to EN ISO 10993 5:2009 [35]. In order to further confirm these findings, the amount of dead cells was estimated using a flow cytometry based SYTOX assay (Figure 5b). Compromised membranes of dead cells allow the fluorescent dye to enter the cell and intercalate into the DNA. After a 3 h incubation with XPC-NP (25 and 100 µg/cm²), only about 6% of the cells were SYTOX-positive, indicating cell death (Table 2). These values are even lower than those found for the untreated control (11.2%). After 24 h incubation with XPC-NP, the fraction of dead cells was found to be only slightly higher (about 9%). Thus, the XPC-NP developed in this work can be considered as non-cytotoxic and safe to use in future extended in vitro/in vivo studies. This finding is in good concordance with previous investigations on the biocompatibility of PS-NP obtained by dialysis of a cellulose phenyl carbamate derivative and of PS coated oxide nanoparticles [26,36].

## 3. Materials and Methods

### 3.1. Materials

Cellulose phenyl carbonates (CPC) and xylan phenyl carbonates (XPC) with defined degrees of substitution (DS) were synthesized by homogeneous conversion of the dissolved polysaccharides (PS) with phenyl chloroformate according to literature [18,19,20]. In brief, the PS were dissolved in an appropriate solvent and a defined amount of phenyl chloroformate was added. Either *N*,*N*-dimethylacetamide (DMA)/LiCl (CPC with low DS < 1.9, XPC with low DS < 0.5) at 0 °C or 1-butyl-3-methylimidazolium chloride/pyridine (CPC with high DS ≥ 1.9, XPC with high DS ≥ 1.5) at 25 °C were employed. The reaction mixtures were stirred for 4 to 24 h and poured into ethanol. The precipitate formed was filtered, washed, and dried under vacuum to yield the final products. DMA and pyridine (anhydrous grade) were purchased from Acros Organics and stored in sealed vessels containing molecular sieves as received. Xylan was provide by Lenzing AG. Cellulose (microcrystalline, Avicel PH-101), 1-butyl-3-methylimidazolium chloride, and all other non-specified chemicals were obtained from Sigma Aldrich (Merck KGaA, Darmstadt, Germany). The polysaccharides were dried at 80 °C and LiCl was dried at 100 °C under vacuum prior to use.

### 3.2. Measurements

Fourier transform infrared (FT-IR) spectra were recorded on a Nicolet iS5 spectrometer (Thermo Fisher Scientific GmbH, Dreieich, Germany) using translucent KBr pellets containing the solid PS samples. Nuclear magnetic resonance (NMR) spectra were recorded at 60 °C in DMSO-d*_6_* (100 mg/mL) with a Bruker Avance I 250 MHz spectrometer equipped with a BBO probe and a Bruker Avance III 400 MHz spectrometer equipped with a BBFO probe (^1^H-NMR: 16 scans; ^13^C-NMR: 8192 to 12,288 scans). DS values were determined by means of ^1^H-NMR spectroscopy according to literature [18]. Trifluoroacetic acid (20 drops) was added to the sample to downfield shift the water related peak.

A Nano ZS zetasizer (Malvern Instruments, Malvern, UK; operating wavelength: 633 nm; detection angle: 173°) was employed to determine the hydrodynamic diameter and polydispersity of the particle dispersions by dynamic light scattering (DLS). The mean particle size was approximated as the effective (Z-average) diameter and the width of the distribution as the polydispersity index (PDI) obtained by the cumulants method assuming spherical shape for the particles. Further details on how the particle size distribution was obtained can be derived from literature [37]. Prior to the DLS measurements, the aqueous particle dispersions were diluted with HPLC grade water (volume ratio: 1:9). Each measurement was performed in consecutive triplicates and the results are presented as the mean values ± standard deviation. 

The scanning electron microscopy images were recorded with a Sigma VP Field Emission Scanning Electron Microscope (Carl-Zeiss AG, Jena, Germany) using the InLens detector with an accelerating voltage of 5 kV. Samples were prepared by placing 10 µL of the NP dispersion on a mica surface that was subsequently dried and coated with a thin layer of gold by sputter coating (Hummer X, Anatech, Sparks, NV, USA).

### 3.3. Preparation and Functionalization of Nanoparticles

The particle dispersions were prepared according to literature [14]. A defined amount of CPC or XPC (10 to 80 mg) was dissolved in DMA (10 mL) and the solution was centrifuged (10 min, 9800 g) to remove dust particles. The optically clear solution was dialyzed (regenerated cellulose membrane, 3500 g/mol molecular weight cut-off) against 1 L of deionized water. The water was renewed five times over the course of two days.

Experiments for functionalization of XPC-NP with dye-605 were performed using particle dispersions with a final mass concentration of 2.7 mg/mL that were prepared as described above with an initial mass concentration of 4 mg/mL. The aqueous XPC-NP dispersions (5 mL) were mixed with borate buffer (5 mL; 0.1 mM, pH value of 8.5) and dye solution (2.5 mL; mass concentration between 1 mg/mL and 0.1 ng/mL), and incubated on a rotary shaker for 24 h at 25 °C. A diluted aqueous solution of ethanolamine (100 mL; 10 vol.%) was added and after 24 h incubation on a rotary shaker at 25 °C the mixture was transferred to a dialysis bag (regenerated cellulose, molecular weight cut-off: 3500 g/mol) and dialyzed against water for 5 days (water was exchanged regularly). A defined amount (1000 µL) was freeze-dried to determine the final mass concentration.

### 3.4. Bio-Compatibility Studies

#### 3.4.1. Cell Culture

The adherent squamous hypopharyngeal carcinoma cell line FaDu (HTB-43, ATCC, Manassas, VA, USA) was utilized for assessing the cytotoxicity of NP. To provide optimal growth conditions and to ensure that potential impairments in cell viability and proliferation are solely due to the presence of NP, cultivation was performed as recommended by the supplier. The cells were cultured in Dulbecco’s modified Eagle’s medium (DMEM) supplemented with 10% (*v*/*v*) fetal bovine serum (FBS; Sigma-Aldrich) [38]. A cell culture incubator (37 °C, 5% CO_2_, 95% relative humidity) was employed for the cultivation. The cells were sub-cultured at a confluence of 80–90%. Therefore, the medium was removed and the cell cultures were washed two times with 5 mL phosphate buffered saline (PBS). Afterwards, 3 mL of 0.05% trypsin-ethylenediaminetetraacetate (EDTA) solution were added and the cell cultures were incubated for 3 min at 37 °C until the cells detached from the culture flask. Cell suspensions were diluted in appropriate ratio by adding fresh medium. In regular intervals, cell cultures were tested regarding mycoplasmic contaminations using the commercial PCR-based Venor Gem mycoplasma detection kit (Sigma-Aldrich).

#### 3.4.2. PrestoBlue Cytotoxicity Assay

After incubation with different particle amounts, the viability of the NP-treated FaDu cell cultures was analyzed using the PrestoBlue assay (Invitrogen, Karlsruhe, Germany). It is based on the ability of metabolically active, vital cells to reduce the non-fluorescent resazurin to the fluorescent resofurin. For that purpose, 15,000 FaDu cells per well in 72 µL of the respective growth medium supplemented with penicillin/streptomycin (P/S) (10,000 U/mL, 10,000 µg/mL) were seeded into a black-walled 96-well plate and cultivated for 24 h. Subsequently, 18 µL NP suspension were added to yield final mass concentrations of NP in the culture medium of 19.4, 97.2, 194.4, and 388.9 µg/mL. This was equivalent to 5, 25, 50, and 100 µg of NP per cm² of the adherent cell layer. The stock PS-NP suspension was diluted with the respective growth medium supplemented with P/S. For the untreated negative control, 18 µL of the respective growth medium supplemented with P/S were applied. As a positive control, 18 µL of Triton X-100 were used, resulting in a final concentration of 0.02% (*w*/*v*). Incubation of the treated cells was performed for 3 or 24 h in a cell culture incubator (37 °C, 5% CO_2_, 95% relative humidity). After the incubation, 10 µL of PrestoBlue reagent were added to each well and the plates were incubated for 30 min in a cell culture incubator at 37 °C. The fluorescence (ex/em: 560/600 nm) was measured with the CLARIOstar microplate reader (BMG LABTECH GmbH, Ortenberg, Germany).

#### 3.4.3. SYTOX Staining

In order to study cytotoxic effects of the NP, SYTOX^TM^ staining was performed. SYTOX^TM^ red dead cell stain (Invitrogen, Karlsruhe, Germany) is a membrane-impermeable DNA dye that is able to enter cells with compromised cell membranes. When binding to DNA, the dye undergoes significant fluorescence enhancement, leading to a selective staining of dead cells when excited with 633/635 nm red laser light, which can be detected at 658 nm.

In order to evaluate potential cytotoxic effects of the NP, FaDu cells (500,000 cells) were seeded into 6-well plates and cultivated overnight. After incubation with the indicated amounts of NP for 3 or 24 h, the cells including the supernatant were harvested by treatment with Accutase (Sigma-Aldrich). After washing twice with 2 mM EDTA solution (in PBS), the cells were resuspended in 500 μL of a 2.5 nm SYTOX^TM^ red dead cell stain solution and incubated at 4 °C in the dark for 15 min. For control purposes, an unstained sample as well as samples containing only NP were prepared simultaneously. Furthermore, a positive control using 0.1% Triton X-100 was included. The samples were measured instantly without performing another washing step, because the dye binds in equilibrium with the DNA and therefore external concentration has to be maintained (FACSCalibur, Becton-Dickinson, Heidelberg, Germany).

## 4. Conclusions

It was demonstrated that xylan phenyl carbonates (XPC) within a broad range of degrees of substitution (DS) self-assemble into well-defined polysaccharide (PS) nanoparticles whereas comparable cellulose phenyl carbonates (CPC) formed particles only in a narrow DS range. The mechanism behind these unexpected differences remains elusive but more in-depth investigation by advanced techniques such as extended small/wide angle X-ray scattering experiments could provide further insight in the self-assembling process of CPC and XPC. Aqueous XPC-NP dispersions showed a high long-term aggregation stability and biocompatibility. Finally, it was possible to directly couple an amino group containing dye to the PS-NP without the need for any laborious activation procedures. The novel reactive XPC-NP are highly valuable for biomedical applications because they can be prepared and functionalized easily and in a modular way. Of high interest for future studies is the coupling of sensitive biomolecules and receptors that can facilitate targeting of specific cells or cellular compartments to enable target specific sensing, imaging, and drug delivery.

## Figures and Tables

**Table 1:** Conditions for and results of the particle formation of polysaccharide (PS) phenylcarbonates with different backbones and degrees of substitution (DS).

ID	PS Backbone	DS_carbonate_	Mass Concentration, [mg/mL]	Diameter, [nm] ^1^	PDI ^2^
**CPC-NP 1**	cellulose	3.00	1	1005 ± 116	0.73 ± 0.04
**CPC-NP 2**	cellulose	3.00	2	2796 ± 571	0.31 ± 0.10
**CPC-NP 3**	cellulose	3.00	4	4015 ± 179	0.54 ± 0.04
**CPC-NP 4**	cellulose	3.00	6	3192 ± 337	0.59 ± 0.16
**CPC-NP 5**	cellulose	3.00	8	4255 ± 684	0.39 ± 0.09
**CPC-NP 6**	cellulose	1.90	1	403 ± 2.4	0.17 ± 0.00
**CPC-NP 7**	cellulose	1.90	2	457 ± 1	0.31 ± 0.00
**CPC-NP 8**	cellulose	1.90	4	862 ± 14	0.31 ± 0.00
**CPC-NP 9**	cellulose	1.90	6	1080 ± 42	0.33 ± 0.00
**CPC-NP 10**	cellulose	1.90	8	1805 ± 136	0.41 ± 0.00
**CPC-NP 11**	cellulose	1.40	1	1332 ± 159	0.64 ± 0.12
**CPC-NP 12**	cellulose	1.40	2	519 ± 8	0.25 ± 0.02
**CPC-NP 13**	cellulose	1.40	4	733 ± 7	0.30 ± 0.02
**CPC-NP 14**	cellulose	1.40	6	864 ± 19	0.33 ± 0.01
**CPC-NP 15**	cellulose	1.40	8	1234 ± 137	0.43 ± 0.01
**CPC-NP 16**	cellulose	1.08	1	226 ± 5	0.13 ± 0.01
**CPC-NP 17**	cellulose	1.08	2	251 ± 2	0.14 ± 0.01
**CPC-NP 18**	cellulose	1.08	4	560 ± 11	0.44 ± 0.07
**CPC-NP 19**	cellulose	1.08	6	345 ± 1	0.21 ± 0.02
**CPC-NP 20**	cellulose	1.08	8	309 ± 2	0.18 ± 0.01
**CPC-NP 21**	cellulose	0.85	1	1443 ± 189	0.47 ± 0.03
**CPC-NP 22**	cellulose	0.85	2	1596 ± 118	0.81 ± 0.04
**CPC-NP 23**	cellulose	0.85	4	1033 ± 321	0.95 ± 0.04
**CPC-NP 24**	cellulose	0.85	6	614 ± 43	0.66 ± 0.08
**CPC-NP 25**	cellulose	0.85	8	977 ± 289	0.82 ±0.09
**XPC-NP 1**	xylan	1.82	1	102 ± 1	0.09 ± 0.03
**XPC-NP 2**	xylan	1.82	2	123 ± 2	0.10 ± 0.00
**XPC-NP 3**	xylan	1.82	4	155 ± 0	0.07 ± 0.01
**XPC-NP 4**	xylan	1.82	6	177 ± 1	0.07 ± 0.00
**XPC-NP 5**	xylan	1.82	8	205 ± 1	0.09 ± 0.01
**XPC-NP 6**	xylan	1.71	1	130 ± 0	0.08 ± 0.00
**XPC-NP 7**	xylan	1.71	2	150 ± 1	0.09 ± 0.01
**XPC-NP 8**	xylan	1.71	4	183 ± 0	0.06 ± 0.01
**XPC-NP 9**	xylan	1.71	6	204 ± 1	0.06 ± 0.02
**XPC-NP 10**	xylan	1.71	8	214 ± 1	0.10 ± 0.02
**XPC-NP 11**	xylan	1.54	1	118 ± 3	0.20 ± 0.02
**XPC-NP 12**	xylan	1.54	2	130 ± 0	0.11 ± 0.00
**XPC-NP 13**	xylan	1.54	4	156 ± 0	0.08 ± 0.01
**XPC-NP 14**	xylan	1.54	6	163 ± 0	0.09 ± 0.00
**XPC-NP 15**	xylan	1.54	8	197 ± 1	0.09 ± 0.01
**XPC-NP 16**	xylan	0.46	1	115 ± 5	0.37 ± 0.03
**XPC-NP 17**	xylan	0.46	2	122 ± 1	0.20 ± 0.00
**XPC-NP 18**	xylan	0.46	4	159 ± 0	0.10 ± 0.02
**XPC-NP 19**	xylan	0.46	6	214 ± 1	0.22 ± 0.02
**XPC-NP 20**	xylan	0.46	8	223 ± 2	0.13 ± 0.02

^1^ Z-average diameter ± standard deviation; ^2^ polydispersity index ± standard deviation.

**Table 2:** Results of the cell death assay (SYTOX assay) on the human hypopharyngeal carcinoma cell line FaDu after incubation with dye functionalized xylan phenyl carbonate nanoparticles (XPC-NP).

Added Component	Percentage of Dead Cells	
	after 3 h	after 24 h
None (positive control)	11.2	n.d.
Triton X-100 (negative control)	99.6	n.d.
XPC-NP (25 µg/cm²)	5.9	8.8
XPC-NP (100 µg/cm²)	6.5	9.3

## Data Availability

Not available.

## Supplementary Materials

The following are available online, Figures S1–S4.

## Sample Availability

Not available.
