# Supplementary material for: Reactive Nanoparticles Derived from Polysaccharide Phenyl Carbonates
Source: Molecules. 2021 Jul 1;26(13):4026. doi: 10.3390/molecules26134026 (PMC8272227; doi:10.3390/molecules26134026)
Supplement: Supplementary file 1 [file molecules-26-04026-s001.zip › molecules-1261693-SI.pdf]

molecules

Reactive Nanoparticles Derived from Polysaccharide Phenyl Carbonates

Supplementary Materials

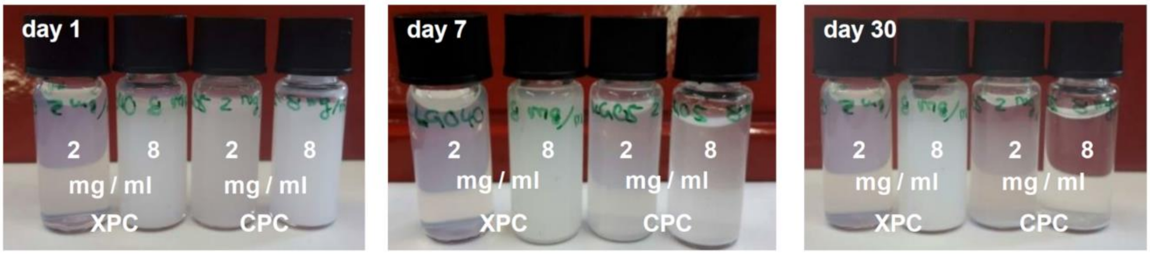

Figure S1. Aqueous particle dispersions obtained by dialysis of solutions with different initial mass concentrations of xylan phenyl carbonate (XPC, degree of substitution of 1.54) or cellulose phenyl carbonate (CPC, degree of substitution of 1.40) in *N*,*N*-dimethylacetamide against water.

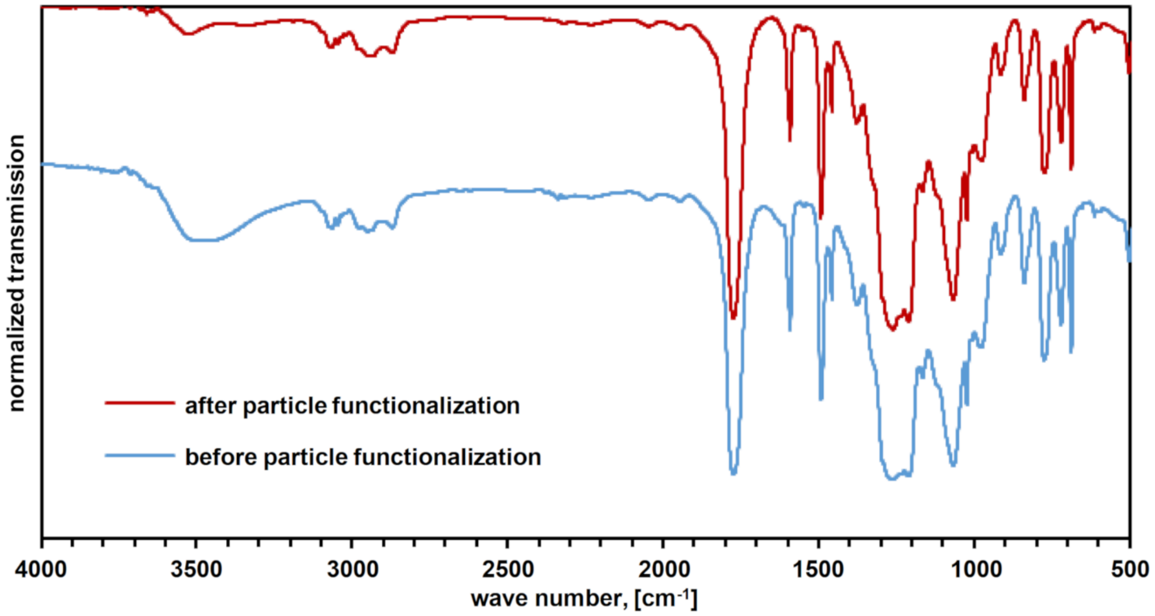

Figure S2. FTIR-spectra of xylan phenyl carbonate (degree of substitution of 1.54) obtained by homogeneous synthesis (blue) and of the same compound after conversion into aqueous particle dispersion (red).

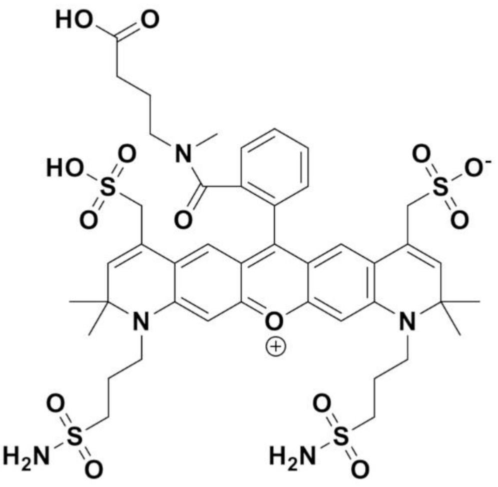

Figure S3. Molecular structure of the chromophore of the dye DY 605 used in this study ($\lambda_{absorption}$ = 600 nm; $\lambda_{emission}$ = 624 nm). The dye features an aliphatic primary amino group that is covalently linked to the chromophore but not shown for confidentiality reasons.

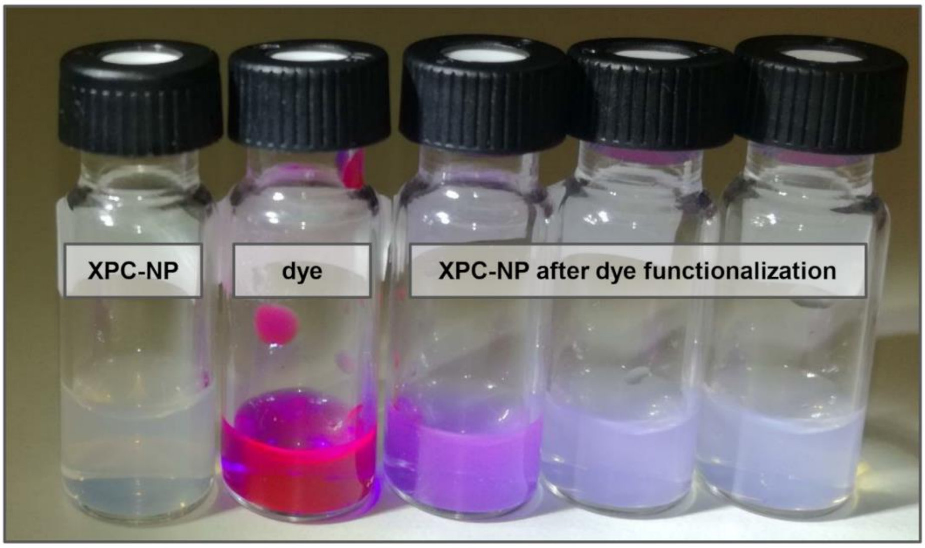

Figure S4. Aqueous nanoparticle (NP) dispersions obtained from xylan phenyl carbonate (XPC; degree of substitution of 1.54, initial mass concentration of 4 mg / ml), aqueous solution of dye DY 605 (1 mM), and XPC-NP dispersions obtained after dye functionalization (initial dye content of 195, 9.77 and 1.95 nm_{dye} per $mg_{XPC\text{-}NP}$).
